# Development of a novel intervention to improve sleep and pain in patients undergoing total knee replacement

**DOI:** 10.1186/s13063-022-06584-3

**Published:** 2022-08-02

**Authors:** K. Whale, R. Gooberman-Hill

**Affiliations:** 1 Musculoskeletal Research Unit, Translational Health Sciences, Bristol Medical School, University of Bristol, Learning and Research Building, Level 1, Southmead Hospital, Bristol, BS10 5NB UK; 2 National Institute for Health Research Bristol Biomedical Research Centre, University Hospitals Bristol and Weston NHS Foundation Trust and University of Bristol, Bristol, UK

**Keywords:** Total knee replacement, Musculoskeletal, Pain, Chronic pain, Sleep, Intervention development, Health psychology, Behaviour change

## Abstract

**Background:**

Up to 20% of patients experience long-term pain and dissatisfaction after total knee replacement, with a negative impact on their quality of life. New approaches are needed to reduce the proportion of people to go on to experience chronic post-surgical pain. Sleep and pain are bidirectionally linked with poor sleep linked to greater pain. Interventions to improve sleep among people undergoing knee replacement offer a promising avenue. Health beliefs and barriers to engagement were explored using behaviour change theory. This study followed stages 1–4 of the Medical Research Council’s guidance for complex intervention development to develop a novel intervention aimed at improving sleep in pre-operative knee replacement patients.

**Methods:**

Pre-operative focus groups and post-operative telephone interviews were conducted with knee replacement patients. Before surgery, focus groups explored sleep experiences and views about existing sleep interventions (cognitive behavioural therapy for insomnia, exercise, relaxation, mindfulness, sleep hygiene) and barriers to engagement. After surgery, telephone interviews explored any changes in sleep and views about intervention appropriateness. Data were audio-recorded, transcribed, anonymised, and analysed using framework analysis.

**Results:**

Overall, 23 patients took part, 17 patients attended pre-operative focus groups, seven took part in a post-operative telephone interview, and one took part in a focus group and interview. Key sleep issues identified were problems getting to sleep, frequent waking during the night, and problems getting back to sleep after night waking. The main reason for these issues was knee pain and discomfort and a busy mind. Participants felt that the sleep interventions were generally acceptable with no general preference for one intervention over the others. Views of delivery mode varied in relation to digital move and group or one-to-one approaches.

**Conclusion:**

Existing sleep interventions were found to be acceptable to knee replacement patients. Key barriers to engagement related to participants’ health beliefs. Addressing beliefs about the relationship between sleep and pain and enhancing understanding of the bidirectional/cyclical relationship could benefit engagement and motivation. Individuals may also require support to break the fear and avoidance cycle of pain and coping. A future intervention should ensure that patients’ preferences for sleep interventions and delivery mode can be accommodated in a real-world context.

## Introduction

In the UK, approximately 100,000 total knee replacement operations are performed annually [1]. Many people who receive knee replacement report improved pain levels and function, but around 20% of patients are dissatisfied with their outcome and experience ongoing pain [2–5]. Long-term pain is linked to decreased function and activity levels and can have a substantial impact on quality of life and wellbeing. In addition, treatment and investigations in relation to ongoing pain are costly to healthcare providers [6]. Pre-operative pain is a strong predictor of pain after knee surgery [7–9]. A recent systematic review and meta-analysis demonstrated that presence of pre-operative pain predicts post-operative pain and that pre-operative pain was one of the most studied predictors of pain after surgery [10]. Cohort research that controls for mediating effects of acute post-surgical pain indicates that patients with high levels of pre-operative pain are more likely to report chronic pain after both total knee and hip replacement [9]. Despite this evidence, there has been limited work designing or evaluating interventions that directly address pre-operative pain. New approaches focused on pre-operative preparation offer a new way to improve pain management and reduce pain severity. Such approaches have the potential to reduce the proportion of people who experience chronic pain after knee replacement.

Pain has a bidirectional association with sleep. The relationship between sleep and pain is supported by a strong evidence base: experimental, cohort, and longitudinal studies have all demonstrated that restricted sleep is causally linked to greater pain [11–16]. Experimental studies have demonstrated that reduced sleep increases the quantity of neurotransmitters related to pain sensitivity and increases inflammatory markers associated with pain reports [17–22]. In addition, reduced sleep increases the subjective impact of pain and negatively impacts individuals’ ability to cope with pain [23, 24]. Longitudinal studies focusing on the effect of sleep on future pain have shown similar results. Results from a Norwegian population-based study show that symptoms of insomnia increase the risk of developing chronic musculoskeletal pain at a 17-year follow-up [25]. Among knee replacement patients, chronic sleeping difficulties and poor pre-operative sleep quality predict post-operative pain [26, 27]. Furthermore, improved sleep has been shown to improve wound healing time, with only modest sleep deprivation causing delays to wound healing [28].

Sleep problems within pre- and post-operative knee replacement patients are well documented. Osteoarthritis (the primary indication for total knee replacement) increases the risk of sleep disturbance, with Osteoarthritis pain linked to lighter sleep. Osteoarthritis is strongly associated with sleep complaints when compared with the general population [29–31]. A key eligibility criterion for total knee replacement is interrupted sleep due to knee pain. Post-operatively, a study of 105 joint replacement patients showed 44–57% of patients experienced disturbed sleep due to night pain [32]. Sleep problems post-surgery are linked to worse functional outcomes. Cremeans-Smith and colleagues [33] found that patients who had undergone total knee replacement and who reported higher pain and sleep disruptions at 1-month after surgery also reported more functional limitations at 3 months post-surgery. Other studies have shown that post-operative sleep disturbances are linked to fatigue, post-operative cardiac events, and poorer mental health [34].

Given the strong relationship between sleep and pain and the impact that sleep problems have on pain response, functionality and quality of life, interventions to improve sleep among people undergoing knee replacement offer a promising avenue. Improvement of sleep in this population has the potential to improve surgical outcomes, healing time, immediate recovery, pain severity, and functional outcomes, as well as to reduce the number of patients who go on to experience long-term pain. For patients who already experience long-term pain or do go on to develop long-term pain, sleep interventions provide a novel patient-centred approach for reducing pain and improving coping.

To date, limited research has been conducted to improve sleep in knee replacement patients, however early work on this shows important promise. A small RCT using a pharmacological intervention (zolpidem vs placebo) demonstrated that improved sleep resulted in significantly decreased post-operative pain and better overall recovery [35]. Although pharmacological interventions show promising results [35], the long-term use of drugs to improve sleep is not desirable or financially optimal. In addition, such drugs decrease non-rapid eye movement and slow wave sleep which reduces the restorative nature of sleep [36]. Non-pharmacological interventions are a more sustainable option for improving sleep in knee replacement patients, are less likely to incur side-effects, and are more cost-effective in the long-term. The development of a non-pharmacological intervention to improve sleep quality and duration in patients undergoing knee replacement has the potential to benefit a wide range of patients and improve long-term outcomes.

Existing sleep interventions offer a good starting point. In this study, we chose to review five sleep interventions used successfully in other populations: cognitive behavioural therapy for insomnia (CBT-i), relaxation, mindfulness, exercise, and sleep hygiene. CBT-i is the most widely used sleep intervention with a strong evidence base [37–39]. CBT-i has been found to be more effective than pharmacological treatments for improving sleep in older adults with short and long-term pain [40]. Relaxation is one of the most common self-help techniques for improving sleep. Previous studies have demonstrated the effectiveness of relaxation for improving sleep [41], with self-relaxation techniques showing improvements in sleep quality for older adults [42]. Mindfulness for sleep has been shown to be an effective long-term sleep intervention [43–45]. Furthermore, for patients with chronic pain, mindfulness can offer improvements in sleep quality, insomnia symptoms, daytime sleepiness, and sleep impairment [46]. Exercise, including resistance training and aerobic exercise, demonstrates promising results for improving sleep quality and quantity in older adults [47, 48]. Meta-analysis results show that exercise improves sleep quality, but may be less effective in improving sleep efficiency [49]. Exercise interventions vary in approaches including weightbearing and non-weightbearing activity, aerobic exercise, resistance training, and strength training. Investigation is needed to assess which approaches are most suitable for knee replacement patients. Sleep hygiene consists of sleep advice and practical information about how to achieve a good sleep environment, for example keeping the bedroom dark, removing electronics. This is the first step to improving sleep but has limited effectiveness as a standalone treatment [49]. Best practice is to combine sleep hygiene with other sleep interventions.

The Medical Research Council’s (MRC) framework for developing robust complex interventions recommends a six-step intervention mapping approach [50, 51] (see Table 1). This study covered stages 1–4 of this process.

**Table 1:** Intervention development steps

1. Define and understand the problem and its causes 2. Clarify which causal or contextual factors are malleable and have the greatest scope for change 3. Identify how to bring about change: the change mechanism 4. Identify how to deliver the change mechanism 5. Test and refine on a small scale 6. Collect sufficient evidence of effectiveness to justify rigorous evaluation/implementation	

To produce a targeted intervention and develop a suitable change mechanism, it is vital to understand the problem and its causes. The existence of sleep problems among knee replacement patients is well documented, but the exact nature of these difficulties is not clear. Sleep problems are varied and each kind may require a different approach to provide benefit. For example, problems getting to sleep (sleep onset latency) will require a different treatment approach than problems with frequent waking. The MRC framework also recommends the use of theory for informing intervention design and delivery [50]. Behaviour change theory is key in successful intervention development and can increase the effectiveness of interventions [52, 53]. Health behaviour change is based on the dual tasks of first initiating and then maintaining change [54]. Initiating a behaviour depends on an individual’s motivations and readiness to change, and their health beliefs. Health psychology models of behaviour change highlight that an individual’s causal beliefs and perceived behavioural control have a direct impact on their willingness to engage in new behaviours [55, 56]. In order to enact successful behaviour change, individuals must feel supported and able to make the change and believe that the new behaviour will make a positive difference [55, 56]. Identifying these beliefs and motivations will result in an intervention specifically designed to meet the needs of the target population and identify any potential barriers to change.

To complete the first stages of the intervention development process, this study explored sleep experiences and patterns within patients undergoing total knee replacement. By exploring sleep experiences of knee replacement patients, we are able to (1) define and understand the problem and its causes, (2) clarify which causal factors are malleable and have the greatest scope for change, (3) identify how to bring about change, and (4) identify how to deliver the change.

## Methods

### Aim

The aim of this study was to complete the initial stages of intervention development to inform the design of a novel intervention to improve sleep and pain in patients undergoing total knee replacement.

### Study design

Qualitative methods were used to complete the first four stages of the MRC intervention development framework. Focus groups were conducted with patients who were about to undergo total knee replacement, and individual telephone interviews were conducted with patients who had undergone surgery and who were still experiencing sleep problems and pain 3 months afterwards. Focus groups were chosen for the pre-operative data collection to foster discussion between participants including to generate any similarities and differences in relation to their sleep issues and views of the sleep interventions. Telephone interventions were chosen for the post-operative data collection to facilitate in-depth reflection about individuals’ experiences of any differences in their pre- and post-operative sleep experiences, and any changes in the acceptability or suitability of sleep interventions between pre- and post-operative phases. Patient and public involvement work indicated that individual (one-to-one) telephone interviews might be more suitable than focus groups with patients after their operation so that individual differences in recovery—such as post-surgical pain and functionality—could be accounted for in practical terms and also in terms of diverse experiences.

### Participants

#### Inclusion and exclusion criteria

Inclusion criteria for pre-operative patients were: currently on the waiting list for a primary knee replacement, aged 18 and over, able to speak and understand English. Patients were excluded if they had been diagnosed with a clinical sleep disorder, currently receiving treatment for a sleep disorder, not experiencing disturbed sleep.

Inclusion criteria for post-operative patients were: at least 3 months post-surgery for a primary knee replacement, experiencing disturbed sleep and knee pain, aged 18 or over, and able to speak and understand English. Patients were excluded if they had been diagnosed with a clinical sleep disorder, currently receiving treatment for a sleep disorder, if they experienced surgical complications resulting in the need for further procedures, or if they have a post-operative infection.

#### Screening

To access eligibility patients were asked to complete a screening questionnaire. Screening included assessments for insomnia (sleep conditions indicators [57], obstructive sleep apnoea (STOP-BANG) [58], and parasomnia and sleep-related movement disorders (individual question). English language fluency was assessed informally through the recruitment phone call. Patients with clinical sleep disorders were not included in this study as clinical sleep disorders require specialist assessment and treatment from existing secondary care services. Post-operative patients were also asked if there were any complications after their surgery, or they had a post-operative infection. Patients with complications or post-operative infection were excluded as pain due to infection or complications requires different treatment approaches and care.

### Recruitment

Patients were identified by Research Nurses from one large urban hospital in South West England. Pre-operative patients were identified from surgical waiting lists. Post-operative patients who had their surgery in the previous 3 to 6 months were identified from reviewing medical records and surgery dates. Patients who had taken part in the focus groups and expressed an interest in taking part in a post-operative telephone interview were also identified from the research team records.

For both pre- and post-operative patients, names and contact details were sent securely to the research team. The research team then sent each patient an information pack by post containing a letter of introduction to the study, a patient information booklet, a screening questionnaire booklet, reply slip, and pre-paid return envelope. Patients interested in taking part in the study were asked to complete the screening questionnaire and reply slip to the study team. The study team contacted each patient by their preferred method (telephone or email) to provide more details about the study and answer any questions. If the patient was willing to proceed, they were invited to attend the next suitable focus groups date (pre-operative patients) or a time was made to conduct the telephone interview (post-operative patients).

### Ethical approval and consent

NHR ethical approval was gained from the South West – Cornwall and Plymouth Ethics Committee in December 2018 (Reference: 18/SW/0281). HRA approval was given in February 2019. Written informed consent was provided by focus group participants. Audio-recorded consent was provided by telephone interview participants due to UK COVID-19 lockdown restrictions.

### Patient and Public Involvement (PPI)

Patient involvement in study design and materials took place through a musculoskeletal patient and public involvement group, many of whom had experience of joint replacement. The group provided input on the topic guides, information booklet, and design and acceptability of the methods.

### Data collection

#### Pre-operative focus groups

Participants were invited to take part in a focus group at the local hospital. Groups were led by the study Chief Investigator (KW) and were audio-recorded using an encrypted digital recorder. A second researcher attended each group to take notes of first utterances to aid transcription, and to assist with practical arrangements. The aims of the focus group were to:

1. Explore sleep experiences and problems within the patient group, including current sleep practices and any methods used to improve sleep
2. Gain feedback on the suitability of existing sleep interventions currently used in other patient populations, and their acceptability for this patient group

Based on previous literature review work, five existing interventions with evidence for effectiveness in other populations were discussed in the focus groups, these were (i) cognitive-behavioural therapy for insomnia, (ii) relaxation, (iii) mindfulness, (iii) exercise, and (iv) sleep hygiene.

#### Post-operative telephone interviews

Interviews were conducted by the first author (KW) and audio-recorded using an encrypted digital recorder. The aims of the interview were:

1. Explore any changes in sleep experiences as a result of surgery
2. Gain feedback on the suitability of existing sleep interventions discussed in the focus groups, and any differences in acceptability and appropriateness pre-operatively versus post-operatively.

### Data analysis

Audio files from focus groups and interviews were transcribed verbatim by an approved external transcription company. Transcripts were anonymised and imported into the qualitative software analysis package NVivo10. Transcripts were analysed using framework analysis [59]. Each transcript was read, and the data was free coded across all transcripts. Early codes were then reviewed in line with relevant behaviour change theory and the MRC guidelines to refine and develop broad themes. Focus group and interview data were coded separately, and then codes compared across both data sets. Commonalities and differences between pre- and post-operative sleep experiences and views on the sleep interventions were identified. Three transcripts were independently double coded by a second qualitative researcher (RGH) and codes were discussed and compared within the team. The aim of this was not to confirm reliability of coding, but to offer further insight and perspective into the data. This brings different approaches and knowledge to the analysis process and enhances rigour [60].

## Results

### Participant demographics

In total, 143 pre-operative and 112 post-operative patients were approached, 43 pre-operative and 19 post-operative patients returned a reply slip, and 39 were excluded for the following reasons: ineligible *n*=11, not able to contact *n*=12, not interested, *n*=7, unable to attend *n*=4, and surgery date moved *n*=5.

A total of 17 participants attended pre-operative focus groups (3–4 participants in each). Groups lasted between 74 and 101 min (mean 85 min). Seven participants took part in a post-operative telephone interview. One participant took part in both the focus groups and an interview and so the total number of participants was 23. Participants were between 3 and 6 months after their surgery, interviews lasted between 53 and 74 min (mean 64 min). Age range was 51–90 years. Thirteen participants reported their ethnicity as white/white British, and one as Afro-Caribbean, nine did not provide this information.

Demographic information is available for focus group participants only. We had planned to collect demographic data for interview participants by postal questionnaire, but this was not possible due to the first UK COVID-19 lockdown from March to July 2020. During the lockdown, non-essential travel was prohibited. We therefore deemed it inappropriate to ask participants to make a trip to the post office or post box to return a demographic questionnaire. We were also unable to collect this data verbally or digitally as there was no ethical approval in place to do so. Table 2 provides a summary of participant demographics.

**Table 2:** Summary of participant demographics

Participant ID	Sex	Group or interview
003	Female	Focus group 1
004	Male	Focus group 1
007	Female	Focus group 1
011	Female	Focus group 1 and interview
015	Female	Focus group 3
016	Female	Focus group 3
017	Female	Focus group 2
018	Male	Focus group 2
022	Female	Focus group 2
023	Female	Focus group 3
025	Male	Focus group 4
027	Male	Focus group 4
028	Male	Interview
029	Female	Focus group 4
030	Female	Focus group 4
036	Female	Focus group 5
040	Female	Focus group 5
041	Female	Focus group 5
107	Male	Interview
111	Female	Interview
113	Male	Interview
115	Female	Interview
118	Female	Interview

### The nature of sleep problems

Participants experienced a range of sleep issues with three key types identified: problems getting to sleep (sleep onset latency), frequent waking, and problems getting back to sleep. Problems getting to sleep caused participants a lot of frustration.

It’s just when my knee’s bad it’s just I can’t seem to find a way to get to sleep – P022

The most irritating thing is when you just think you are about to nod off and you don’t, you come back again and that can happen several times – P027

One participant described feeling so frustrated that they wanted to scream.

In recent years it’s got worse, sometimes I just want to scream because I want to go to sleep and I toss and I turn and I end up getting up and going to the loo and taking some pain killers because that might help – P022

In some cases, participants were awake for several hours.

I saw 2am go round last night. Tried everything. I went to bed, I felt really tired and I thought I can’t. You are trying to lie and you don’t know which way to go. I think I read and I put the light off about 1.45am – P030

Other participants had problems in both getting to sleep and with waking during the night.

It’s just getting to sleep, and not staying asleep. Because I can’t stay asleep – P040

Waking during the night was the most common problem discussed by participants. Many were waking multiple times causing both poor quality sleep and reduced total sleep time.

Last night I work up four times…not to go to the loo or anything like that, just because my knee was playing up – P004

I could have four hours’ sleep but in that four hours I could wake every hour. I try not to look at the clock but it’s physically impossible because you see it when you go back and then it takes you a while to go back to sleep. I hate it. – P030

Getting back to sleep was an issue for many participants.

During the night if I do wake up, I do get a little bit iffy cause I can’t go back to sleep straight away – P004

[I have] no problems going off, but then I am waking up after about four or five hours and I struggle to back to sleep – P025

### Causes and strategies for sleep and pain

Participants identified a number of different causes for their sleep problems. As expected, the most common cause of sleep problems was knee pain and discomfort; and participants talked about how this manifested itself and identified factors that impacted their pain as well as strategies they used to reduce pain and enhance sleep.

It’s the pain basically, always you know, take that away and I can sleep like a log – P004

Sleep position and positioning of the knee were frequently discussed. Some participants had found they could not sleep in their preferred position, for instance, side sleeping, as this put too much pressure on their knee joint.

I don’t like sleeping on my back … [on your side] then you put one knee onto the other one … that doesn’t work either and you try and cross them over - P027

When my knees are bad I have to lie on my back. I can’t lie on my side because they are so swollen – P029

The need to support the knee to relieve pressure was also highlighted. Participants described using rolled-up towels or pillows to relieve pressure on their knee.

“Like a towel rolled up, if I put in under my, the back of my knee, if you can sleep like that it gives you a lot of relief” – P004

“The thing that would help me would be a pillow, like I said, I use between my knees” – P004

Frequently experiencing night pain had led to some participants feeling anxious about their sleep and about potential pain and disruption before it had happened. One participant talked about using classical music to help distract themself.

Classical music doesn’t help me sleep but it stops you thinking about all the other things going through your mind. Or wondering if this [the knee pain] is going to keep me awake tonight again – P030

Having a ‘busy mind’ was repeatedly talked about. Many participants found that either when initially going to sleep or when they were woken up, they had trouble quieting their mind.

[my sleep] is very restless, restless legs, restless mind, all sorts of things. Anything out of the blue you know, the least little thing sometimes you just thinking about – P007

I got for a solid four hours, then you have got all these different things you are thinking about. Sometimes, it is alright and when you go back. You just lie there hoping and waiting to go back to sleep – P036

### Views about existing sleep interventions

Five existing sleep interventions were discussed: cognitive-behavioural therapy for insomnia (CBT-i), exercise, relaxation, mindfulness, and sleep hygiene. Intervention preferences were highly individualistic with no single intervention being preferred over the others by all participants. No differences in pre- or post-operative suitability were raised. Views on sleep hygiene were limited to disclosure of any techniques or sleep aids participants had tried.

#### Cognitive behavioural therapy for insomnia

CBT-i particularly appealed to participants who wanted to understand more about why they were experiencing sleep problems.

The structured programme that helps you identify and replace thoughts and behaviour across the worst of your sleep problems. I would like to find out what is causing my sleep problems. – P029

The language used to describe CBT-i had an impact on the participants’ perception of effectiveness and their interest in engaging with this option. Some participants found the language used to describe CBT-i difficult to understand.

Probably I just didn’t understand it. I haven’t given up on it … There’s just some ambivalence involved with it – P027

Passive descriptions of CBT-i such as ‘talking therapy’ was off-putting for some participants.

I can’t see that talking about it is going to do any good to be quite honest. I might be wrong, but I am not sure – P036

I don’t see where talking about it will help, because I’m not worrying about any particular thing. I don’t see where that would be helpful to me – P029

However, language related to participants taking a more active approach, such as ‘training’ and ‘structured programme’ were perceived more positively.

If I could train myself to stop thinking about why I have woken up with the pain… that would be really good – P030

Well a structured programme and no sleeping pills. I am interested in that – P036

#### Exercise

All participants viewed exercise positively. However, discussions about exercise focused on knee function and pain rather than sleep.

I do agree with exercise because when I play golf … sometimes I can be in agony the day after [but] my muscles are a lot better – P007

[discussing weekly exercise class] I have found that if I do the exercises it does actually help after a while, you seem to sort build the muscle up – P111

Some participants felt that while exercise was beneficial it would not be appropriate for them as they were in too much pain. Ensuring the exercises were appropriate for their health condition and functional limitations was highlighted as being important.

See I love the idea of Pilates but a lot of it is on the floor. You’d get me on the floor but you’d never get up me up again! [laughter] – P023

[discussing a gym class] you’re supposed to lift that leg out and stretching till, well, you get that leg up in the air, what happens with the other leg? – P003

#### Relaxation and mindfulness

Participants thought that relaxation and mindfulness were similar in their approaches and techniques and so we describe opinions about these methods together. Participants who had experience of using mindfulness techniques reported finding them helpful.

[My Doctor] put me onto this what was it called, it was really quite helpful actually. Like mindfulness and you could have different things each day … he did give me app actually … it was quite helpful and quite distracting.… I think it was five minutes, ten minutes, you could choose – P115

Participants discussed a range of relaxation techniques that they had either tried or were interested in trying. The intuitive nature of these techniques was appealing.

If you get into bed and you’re puffing you’ll never go to sleep, no you’ve got to calm your breathing down … breathe through your nose nice and steady – P018

I do try like muscle relaxing, especially when I’m lying there. I’ll think to myself, right, start with your toes.… I do find it quite relaxing, um, sort of mind over matter a little bit – P011

Relaxation also appealed as the techniques were simple to learn, especially with some initial guidance.

It sounds like something I could do. It is pretty straightforward if somebody helps you initially with it – P027

#### Barriers to engagement

Barriers to engaging in all the interventions were discussed with all participants. Key barriers identified were motivation and the need for support, belief in the efficacy of the intervention, and tackling the pain before sleep.

A number of participants said that whilst they were interested in trying to improve their sleep, they would benefit from having someone motivate and encourage them.

I do find things if I’ve got somebody to help me through it I do much better than just trying to cope with doing something on my own. I just need that motivation –

If you haven’t got somebody saying to you, hey come on it’s 10 o’clock, let’s [do] your whatever, you’re there and you’ve got to motivate yourself to do it and you’re tired, you know and you think, I can’t be bothered – P003

Belief in the efficacy of the sleep interventions and understanding how they worked was a substantial barrier for some participants. One participant who woke regularly in the night felt that actively engaging in a behavioural technique to get back to sleep was counterintuitive.

Once you’re awake you’re looking forward to going back to sleep and I think going through the motions of doing these exercises of muscle [relaxation], breathing exercises, it’s waking you up rather than putting you to sleep – P004

Another felt resigned that nothing would help them to sleep better.

I can’t think anything would help me. I don’t know, I don’t think they would help me – P007

A fundamental barrier to engaging in the sleep interventions was the belief that the pain needed to be directly targeted rather than sleep. A small number of participants raised doubts that improving sleep would make any difference to their knee pain.

I mean all of us, once you get pain in your knee when you wake up, all you want to do is get rid of that pain and I can’t see any of this getting rid of the pain – P004

These participants saw waking in the night due to knee pain as inevitable. The only solution for improving sleep was to eliminate the pain, rather than using techniques to improve sleep to help manage the pain.

I’ve looked at both of these papers [descriptions of the sleep interventions] and I’ve listened to what you’ve said there, I can do all the relaxation, go off to sleep very quickly, but as soon as that [the knee] kicks in, it wakes you up – P004

Participants’ health beliefs and their framing of the sleep and pain relationship was key to levels of engagement. Framing pain and sleep issues as a one-way causal relationship (pain causes disturbed sleep), rather than as a bidirectional/cyclical relationship (disturbed sleep can also cause increased pain), led to a lack of engagement.

I don’t have any problems sleeping, it’s just the pain wakes you up – P017

Being in pain was also seen as a barrier to engagement. One participant described how being in pain would prevent them from trying any of the sleep techniques, such as breathing or muscle relaxation.

But if you’re in a lot of pain, that’s a different thing. How can you get out of pain to do that? – P007

### Mode of intervention delivery

Exploration of the delivery of the change mechanism focused on participants’ preferences for access to existing sleep interventions. Discussions covered three key areas: digital versus non-digital, remote versus face-to-face, and group versus one-to-one.

#### Digital versus non-digital

Views on using digital interventions such as smartphone apps or websites were divided. Those participants who owned a smartphone or tablet and already used apps said they would feel happy trying digital options. Digital options appealed due to being able to engage in the intervention in their own time, and that no travel was needed.

I would probably like the App. It would be in my own time. That is probably the main thing. Sooner or later I will be back at work. I am filling my time at the moment with things like this, things that might be useful. I definitely would try and App first. – P027

If I could do it online if they could help that way probably easier than me having to go somewhere – P022

Other participants reported that they were not confident to use digital technology and that digital-only options would create a major barrier to engagement.

The only thing for me, I can’t go online. I wouldn’t have a clue. I mean that seriously. I wouldn’t have a clue how to get online or anything like that. – P030

Definitely a barrier for me. I wouldn’t have a clue. I just about know how to open my phone and only because my son teaches me, because he is worried. – P030

#### Face-to-face versus remote

Views on face-to-face or remote delivery also varied between participants. Many participants said that they would prefer to see someone in person.

I think face-to-face … online it’s okay but it’s a bit impersonal I think – P011

I’ll tell you what would be good, if I could be in a room with someone and they actually went through it with me … then once I’ve got it I would be able to go away and do it for myself – P111

A key consideration was location and travel. Long travel time or difficulties with parking were highlighted as barriers to attending face-to-face appointments.

It would depend where the one-to-ones were. Otherwise I’d have to do it online because coming across here in the morning was fine, but to do it regularly would be … I plan for a few days to come across here – P023

As long as the group was somewhere that was easily accessible to people. I find things are out of the way, like [local area] it’s difficult to get to – P111

Due to the timing of the telephone interviews during the first UK COVID-19 lockdown, one participant reflected on initial preference versus waiting time.

I think face-to-face would be better actually … but with lockdown and shielding so if it meant waiting for a long time then maybe online then – P115

#### Group versus one-to-one

Participants also discussed whether they would prefer a group format or individual. Views varied depending on the type of intervention being discussed with a strong preference for group delivery for exercise interventions. Group sessions also appealed due to the group support and shared experience. One participant reflected on their experience of a fitness group they attended.

it’s quite helpful, actually, because we’re all in the same boat and, er, we all mix quite well.… There are all sort of ages and states of fitness and so on and, um, we just get on quite well and we help one another. Um, we’re almost a team in the same boat together – P028

Some participants reported feeling more motivated in a group setting.

I think it encourages you more to – like, when you’re on your own you think, oh have I got to do it, you know, but when you’re in a group, you know, especially if you’ve got like a teacher or a person who’s running the group er says ‘Come on, you’ve got to do another one’ you know. – P011

## Discussion

Patients who were waiting to have their knee replacement experienced a range of sleep problems. The key problems were difficulty getting to sleep (sleep onset latency), frequent waking at night, and difficulty getting back to sleep after night waking. Participants said that the main reasons for these issues were knee pain, knee discomfort, and having a ‘busy mind’. Participants also thought that all the sleep interventions described to them in the study were generally suitable and acceptable with no differences identified between the pre- and post-operative groups. Overall, there was no preference for one existing sleep intervention above another. Participants had divided views for and against digital delivery such as apps or websites, and the variety highlights a need for personalisation and tailoring in intervention delivery.

Our results build on existing literature about sleep problems in this population. Sleep complaints reported in previous studies mirror the results of this work, with sleep latency, duration, and sleep fragmentation all commonly reported [61]. The range of sleep issues and intervention preferences support the need for a tailored intervention approach. Tailoring the type of sleep intervention and the mode of delivery to the individual means that there will be a higher degree of autonomy (having a sense of choice) and competence (feeling able to perform a task). These are important mediators of behaviour change and intrinsic motivation [62]. Supporting an individual to improve their sleep through a behavioural intervention requires the individual to feel motivation first to engage in, and then to sustain the behaviour. Self-determination theory (an established theory of motivation) has demonstrated that developing a sense of autonomy and competence is critical to enable individuals to self-regulate and sustain behavioural change [56]. Treatment environments that enable autonomy and support patients’ confidence are more likely to enhance concordance and positive health outcomes [54]. Taking a tailored approach to improving sleep in pre-operative knee replacement patients by providing a choice of existing sleep interventions and delivery modes is therefore more likely to result in patient engagement and longer-term change.

To enact successful behaviour change it is also vital to understand any perceived barriers to engagement. A notable barrier identified in this work was the health belief that pain needed to be targeted before sleep and that there was a one-way causal relationship between pain and sleep, as participants thought that pain influenced sleep and not that sleep influenced pain. The common sense model of health and illness [55] suggests that perceived causes of a condition and the curability or controllability form part of an individual’s illness perception. This then impacts how someone responds to treatment recommendations. If pain and sleep are thought to relate to each other in a one-way causal relationship such that disturbed sleep is seen as an inevitable consequence of knee pain, then individuals are unlikely to feel they have any control over their sleep without first addressing and reducing their pain. This view also externalises locus of control which is linked to a lack of treatment engagement and concordance [63]. External locus of control removes autonomy and self-efficacy from the individual and positions them as passive in relation to their health experiences. This view therefore removes any motivating factors to engage in active treatments or behavioural change.

Having pain featured as a barrier to engaging in an intervention. The fear avoidance model of pain [64], which is underpinned by a biopsychosocial approach, offers insight into why this may be. The key components of this model are hypervigilance and pain catastrophizing. Individuals who engage in pain catastrophising experience greater pain-related fear. This leads to pain avoidant behaviours and pain hypervigilance: an excessive focus on the pain experience and bodily sensations of pain [65]. This can also result in ‘kinesiophobia’, an irrational and paralysing fear of injury and movement. Individuals who are in the fear-avoidance cycle of pain, therefore find it very difficult to focus on anything other than their pain or engage in activities which may break this cycle [66, 67]. Intense pain, such as night pain that causes waking, also decreases the ability to perform cognitive tasks due to difficulties in disengaging attention from the pain experience [68, 69].

Results of this study identify key components of patients’ health beliefs that an acceptable and effective intervention for improving sleep would need to address. It may be necessary to target certain health beliefs and maladaptive coping strategies to support patient engagement. Tailoring a sleep intervention to individual patient needs and preferences will also result in greater engagement and acceptability. The mode of intervention delivery also requires flexibility, particularly in relation to digital and non-digital options, and face-to-face and remote options. Future interventions should be designed to be adaptable to the individual’s needs and preferences, whilst remaining feasible to delivery within a real-world context.

## Strengths and limitations/weaknesses

This study had a small but robust sample size. Due to COVID-19 restrictions, recruitment was closed early. However, depth of discussion in focus groups and interviews was good, with high quality dialogue. Analysis was conducted to a high standard with a subset of data double coded to enhance rigour.

We included participants who spoke English with sufficient fluency to engage in a focus group or interview. No participants were excluded on this basis however study invitation and recruitment materials were only provided in English. It is important to acknowledge this limits the transferability of findings as further work would need to ensure that potential interventions are accessible in other languages. As described in the methods, it was not possible to collect demographic data from the interview participants. Although it would have been preferable for us to have been able to fully describe the sample, the absence of the information does not undermine the veracity of the findings as a comparison on the basis of age or ethnicity was not an aim of the study. However, we would like to have been more confident about whether the sample appropriately reflected the sociodemographic diversity of patients undergoing knee replacement. Further intervention development would need to address this important issue so that an intervention is inclusive and appropriate for all patients. Patients with clinical sleep disorders were excluded, this was because clinical sleep disorders require specialist assessment and treatment from existing secondary care services. Post-operative patients who experienced surgical complications or a post-operative infection were excluded as pain due to infection or complication requires different treatment approaches and care.

Pre-operative focus groups were conducted face-to-face at a local hospital site. This may have meant that individuals with limited resources to enable them to travel or who could not travel independently were less likely to take part. To reduce the chance that this would happen we offered travel expenses, free parking spaces next to the building, and were able to arrange and pay for taxi services if needed. Approximately half of the participants were reimbursed for public transport costs and two were provided with a taxi.

Post-operative telephone interviews were conducted in 2020, during the first UK lockdown in the COVID-19 pandemic. During this time, evidence suggests that sleep problems rose in the general population, with survey data estimating 50% of the UK population had more disturbed sleep than usual and 39% reporting sleeping fewer hours per night [70]. This could have impacted the findings on post-operative sleep experiences by exacerbating sleep issues related to knee pain and surgical recovery. However, we found no differences between pre- and post-operative views on the existing sleep interventions.

After completion of this work new Medical Research Council guidelines on intervention development have been published [71]. These guidelines provide additional elements relating to the context of intervention development, diversity in stakeholder views, key uncertainties, and resource and outcome consequences. The new guidance raises further reflection points for the next phase of intervention development work and will help to ensure that a diverse range of perspectives and approaches are used in the intervention design and conduct.

## Conclusion

The study completed the initial stages of intervention development to develop a novel intervention to improve sleep and pain in patients undergoing total knee replacement, following the current MRC framework. Key barriers to engagement related to participants’ health beliefs, which included viewing pain and sleep as a one-way causal relationship, and pain fear avoidance. Addressing beliefs about the relationship between sleep and pain and enhancing understanding of the bidirectional/cyclical relationship could benefit engagement and motivation. Individuals may also require support to break the fear/avoidance cycle of pain and coping. A future intervention should ensure that patients’ preferences for sleep interventions and delivery mode can be accommodated in a real-world context and these include offering both digital and non-digital options and tailoring for face-to-face or remote preferences. As part of an intervention, provision of appropriate support to address health beliefs and unhelpful coping strategies may increase engagement, concordance and benefit.

## Data Availability

Supporting data can only be made available to bona fide researchers subject to a data access agreement and following approval from the University of Bristol Data Access Committee. Data are available on request at the University of Bristol data repository, data.bris, at 10.5523/bris.n8gd8ekdu3272s1kdgq4caons.
